# Response to: Comment on “Soluble Urokinase-Type Plasminogen Activator Receptor Plasma Concentration May Predict Susceptibility to High Altitude Pulmonary Edema”

**DOI:** 10.1155/2018/8036759

**Published:** 2018-05-02

**Authors:** Matthias Peter Hilty, Stefanie Zügel, Michele Schoeb, Katja Auinger, Christoph Dehnert, Marco Maggiorini

**Affiliations:** ^1^Medical Intensive Care Unit, University Hospital of Zurich, Zurich, Switzerland; ^2^Institute for Sports Medicine, Rupert-Karls University of Heidelberg, Heidelberg, Germany

We would like to thank G. Sikri and S. Bhattachar for their insightful comment [1] on our article suggesting a role of low-grade cellular-based inflammation as indicated by elevated soluble urokinase-type plasminogen activator receptor (suPAR) plasma concentrationon in the pathogenesis of high altitude pulmonary edema (HAPE) [2].

The adaptation of the cardiovascular system to hypobaric hypoxia is an important entity in all high altitude studies. Recent data from a study using pharmacological blockade of the sympathetic and parasympathetic nervous system [3] suggest that even though sympathetic nervous system activity increases as compared to normoxic state, parasympathetic withdrawal rather than sympathetic activation seems to be the cause of the increase in heart rate observed in healthy humans subjected to hyperbaric hypoxia. Even though the effect of dexamethasone on the autonomic nervous system is not fully understood, dexamethasone administration has previously been shown to decrease heart rate during hypoxic exposure [4]. It is thus in concordance with current literature that dexamethasone intake is the likely cause of the different course of HR during hypoxic exposure seen in our study population (*Δ*HR +16/min versus −7/min, *p* < 0.01, in the *no prophylaxis* (*n* = 31) versus the *dexamethasone prophylaxis* (*n* = 10) group).

As G. Sikri and S. Bhattachar point out, it is empirically known that HAPE incidence at altitude can only be assessed after several days of high altitude exposure. We agree, epidemiological studies have clearly shown that HAPE is a hydrostatic type of pulmonary edema which develops within the first days but no later than five days of exposure to high altitude. In the setting of the Margherita studies, we observed that up to 70% of the HAPE susceptible mountaineers develop HAPE within 72 hours of arrival [5, 6]. Results of a recent study of hemodynamic adaptation to a constant altitude of 3454 m over three weeks further suggest the occurrence of early pulmonary vascular remodeling within less than 21 days as an underlying mechanism for a subsequent decrease in HAPE incidence [7]. Our study was designed to answer the question whether low-grade cellular-based inflammation as indicated by elevated suPAR plasma concentration could predict HAPE susceptibility. Even though our data indicates such a connection, the question if suPAR plasma concentration also correlates with HAPE incidence at altitude remains unanswered. As described in our study, after the first 24 hours at altitude, subjects that have not received dexamethasone prophylaxis before ascent were randomized into a group that received *post exposure* (“late”) *prophylaxis* of 8 mg oral dexamethasonebidaily (*n* = 20) and a *control* group that did not receive dexamethasone (*n* = 11). Thus, HAPE incidence at high altitude depended on dexamethasone administration, and the groups are not large enough for a subgroup analysis regarding baseline suPAR plasma concentration. It is however of interest that dexamethasone application at high altitude partly reversed the hypoxia-associated increase in suPAR and interleukin-6 (IL-6) plasma concentration within 48 hours, while this was not the case for c-reactive protein (CRP; see Table 1).

## Figures and Tables

**Table 1:** Development of suPAR, CRP, and IL-6 plasma concentration during 48 hours of exposure to 4559 m.

	High altitude, 24 hours	High altitude, 72 hours	*p* value, delta median (95% CI)
*suPAR [ng/ml]*			
Entire sample (*n* = 31)	2.27 (2.20)	2.08 (2.00)	*p* = 0.34, Δ0.10 (−0.05–0.20)
No steroids (*n* = 11)	2.13 (2.00)	2.03 (1.90)	*p* = 0.34, Δ-0.15 (−0.35–0.10)
Therapy (*n* = 20)	2.33 (2.25)	2.11 (2.05)	*p* = 0.04, Δ0.20 (0.00–0.30)
*CRP [mg/l]*			
Entire sample (*n* = 31)	3.57 (2.20)	3.43 (2.60)	*p* = 0.77, Δ0.10 (−0.75–1.40)
No steroids (*n* = 11)	1.81 (1.60)	2.76 (2.60)	*p* = 0.08, Δ-1.13 (−2.70–0.05)
Therapy (*n* = 20)	4.45 (2.50)	3.76 (2.50)	*p* = 0.18, Δ1.25 (−0.30–2.40)
*IL-6 [ng/l]*			
Entire sample (*n* = 31)	2.71 (2.05)	1.51 (0.73)	*p* = 0.01, Δ1.04 (0.23–2.60)
No steroids (*n* = 11)	2.45 (2.05)	2.67 (1.75)	*p* = 0.58, Δ0.33 (−3.19–1.25)
Therapy (*n* = 20)	2.91 (2.21)	0.64 (0.34)	*p* = 0.01, Δ2.18 (0.69–4.06)

Comparison of inflammatory marker plasma concentration at 24 versus 72 hours was performed using related sample Wilcoxon signed rank test. Values are given as median (IQR). Delta median corresponds to the median of the difference between samples from both timepoints. suPAR: soluble urokinase-type plasminogen activator receptor; IL-6: interleukin-6; CRP: c-reactive protein; CI: confidence interval.
